# Organ-specific safety profile of bioinspired short antimicrobial peptides in zebrafish embryos

**DOI:** 10.3389/fphar.2025.1593683

**Published:** 2025-05-27

**Authors:** Sahar Isa Da’as, Hajira Afreen, Aseela Fathima, Ahmad M. Hani, Nura A. Mohamed, Md Mizanur Rahman, Patrick G. Burgon, Sergio Crovella, Haissam Abou-Saleh

**Affiliations:** ^1^ Research Department, Sidra Medicine, Doha, Qatar; ^2^ College of Health and Life Sciences, Hamad Bin Khalifa University, Doha, Qatar; ^3^ Biological Science Program, Department of Biological and Environmental Sciences, College of Arts and Sciences, Qatar University, Doha, Qatar; ^4^ Biomedical Sciences Department, College of Health Sciences, Qatar University, Doha, Qatar; ^5^ Biomedical Research Center, QU Health, Qatar University, Doha, Qatar; ^6^ Department of Chemistry and Earth Sciences, College of Arts and Sciences, Qatar University, Doha, Qatar; ^7^ Laboratory of Animal Research Center (LARC), Qatar University, Doha, Qatar

**Keywords:** bioinspired short antimicrobial peptides (BSAMPs), zebrafish, developmental toxicity, safety, organ-specific toxicity

## Abstract

**Objective:**

Antimicrobial peptides (AMPs) are key components of the innate immune system, exhibiting broad-spectrum antibacterial and immunomodulatory activities. Building on these properties, we designed bio-inspired short antimicrobial peptides (BSAMPs) using computational and bioinformatics approaches. Following promising *in vitro* results demonstrating selective anticancer activity against colorectal cancer cells, this study aimed to investigate the *in vivo* organ-specific safety and toxicity profiles of two selected BSAMPs—Peptide C (GVLCCGYRCCSKWGWCGTT) and Peptide E (CWWMTRRAWR)—using the zebrafish model.

**Method:**

Zebrafish embryos were exposed to various concentrations of Peptide C and Peptide E. Phenotypic toxicity endpoints—including Lethal Concentration 50 (LC_50_), cardiotoxicity, neurotoxicity, and hepatotoxicity—were assessed.

**Result:**

The LC_50_ values for Peptide C and Peptide E were determined to be 162.2 μg/mL and 131.82 μg/mL, respectively. Peptide C caused minimal cardiovascular effects below 150 μg/mL but induced neurotoxic and hepatotoxic effects at concentrations exceeding 100 μg/mL. Peptide E exhibited developmental toxicity at concentrations above 100 μg/mL, along with cardiotoxic effects such as reduced heart rate, variable locomotion patterns, and clear hepatotoxic responses.

**Conclusion:**

This study highlights distinct organ-specific toxicity profiles for Peptides C and E and underscores the importance of careful preclinical evaluation of BSAMPs. The zebrafish model provided valuable insights into the potential safety concerns of these peptides, supporting their further investigation and refinement for future therapeutic development.

## 1 Introduction

Antimicrobial peptides (AMPs) are small, positively charged peptides widely produced by many life forms. They possess antibacterial properties, capable of preventing bacterial growth by interacting with microbial cell membranes. AMPs are attracted to negatively charged microbial membranes due to their positive charge. Upon reaching the membrane, AMPs insert themselves into the lipid bilayer, causing disruptions that compromise membrane integrity. This disruption can occur through pore formation, increasing membrane permeability and leading to cell lysis, or through a “carpet model” where peptides spread across the membrane surface, causing it to break apart (Bui Thi Phuong et al., 2024). Some AMPs penetrate the cell membrane, interfering with critical intracellular functions and inhibiting essential cellular processes. This multifaceted mode of action makes AMPs effective against a wide range of pathogens, including bacteria, fungi, and viruses, thereby reducing the likelihood of resistance development compared to traditional antibiotics. Recent research has revealed that AMPs can also disrupt biofilms, protective layers formed by bacterial communities that are highly resistant to conventional treatments. By targeting both free-floating (planktonic) cells and these biofilm structures, AMPs offer a promising solution against multidrug-resistant infections (Mehraj et al., 2024).

Several AMPs warrant attention, including human LL-37, M2163, and M2386 from the bacterium *Lactobacillus casei*, and Pn-AMP1 and Pn-AMP2 from the plant *Pharbitis nil*. Interestingly, plants, particularly extremophiles, boast a higher abundance of AMPs compared to other eukaryotes (Tsai et al., 2015; Morizane et al., 2012; Mignone et al., 2022). This adaptation allows them to thrive in harsh environments and combat diverse pathogens (Benko-Iseppon and Crovella, 2010; Benko-Iseppon, 2020; de Jesús-Pires et al., 2020). The rich source of AMPs in plants, especially extremophiles, has fueled research into their potential for tackling infectious diseases. Functioning as a core component of the innate immune systems in both plants and animals, AMPs defend against infections through various mechanisms, including immunomodulation and membrane disruption (Perez-Rodriguez et al., 2022; Sowińska et al., 2018). Their broad-spectrum effectiveness against pathogens stems from their multifaceted modes of action, which also diminish the risk of microbial resistance development. As natural components of the immune system, AMPs generally pose a lower risk of side effects compared to antibiotics, highlighting their growing importance in clinical and medical applications. Looking beyond their antimicrobial properties, AMPs hold immense potential as anti-cancer agents. They can selectively target and disrupt cancer cell membranes, induce apoptosis through membrane disruption, interact with intracellular targets, and modulate the immune response, offering a multifaceted approach to cancer treatment (Piktel et al., 2024).

Despite their broad-spectrum efficacy and natural origin, the safety profile of AMPs remains a significant consideration for clinical development. Although AMPs generally pose a lower risk of resistance development compared to traditional antibiotics, their potential cytotoxicity, hemolytic activity, and immunogenicity must be carefully evaluated (Tsai et al., 2015; Di et al., 2020; Oyama et al., 2022). Some AMPs can inadvertently disrupt mammalian cell membranes due to similarities in lipid composition, leading to adverse effects (Mahlapuu et al., 2016; Lei et al., 2019). Furthermore, systemic administration may trigger undesired immune responses or organ-specific toxicities. Comprehensive *in vitro* and *in vivo* toxicity profiling has been increasingly recognized as essential to the clinical development of AMPs to ensure selective targeting of pathogenic cells while minimizing harm to healthy tissues, as emphasized in our previous work (Hashem et al., 2025) and corroborated by broader studies (Mahlapuu et al., 2016; Lei et al., 2019; Magana et al., 2020). Thorough assessment of AMP safety, including evaluations of hemolytic potential, cytotoxicity toward normal cells, and organ-specific effects in animal models, is essential to advance these promising molecules toward clinical application. However, translating AMPs into clinical applications presents several hurdles. Challenges include unknown pharmacodynamics (Fox, 2013), structural instability, susceptibility to enzymatic degradation (Mookherjee et al., 2020), and cytotoxicity (Fjell et al., 2012). Natural extraction from plants or animals is equally problematic – it’s time-consuming, yields are low, and the extracted AMPs often have a short half-life and hemolytic activity. To address these limitations, researchers have turned to advanced high-throughput molecular and bioinformatics tools. These sophisticated techniques, utilizing *in silico* methods, enable the design of novel, short Bio-inspired Antimicrobial Peptides (BSAMPs). By incorporating chimeric molecules and establishing structural scaffolds, this approach aims to optimize the properties of these potential therapeutics for future clinical use (Lei et al., 2019; Santos-Silva et al., 2021).

Zebrafish (*Danio rerio*) are unique animal models widely used in biomedical research for studying developmental and physiological processes, particularly at very early stages of development (Teame et al., 2019; Abou-Saleh et al., 2019; Al-Ansari et al., 2022; Al-Jamal et al., 2020; Al-Kandari et al., 2019; Dooley and Zon, 2000; Nasrallah et al., 2018; Stainier and Fishman, 1994; Al-Asmakh et al., 2020). This remarkable similarity extends to functionality, with 84% of human disease genes having zebrafish counterparts (Zakaria et al., 2018; Cassar et al., 2020). This high degree of conservation in cellular morphology, genetics, and physiology makes zebrafish an excellent model for studying various human diseases and disorders such as cancer, cardiovascular disorders, kidney disorders, neuromuscular disorders, digestive disorders, liver disorders, diabetes, and infectious diseases (Teame et al., 2019; Howe et al., 2013).

We recently developed two novel broad-spectrum antimicrobial peptides (BSAMPs), Peptide C (GVLCCGYRCCSKWGWCGTT) and Peptide E (CWWMTRRAWR), protected under invention QU2024-009 and U.S. disclosure Docket Number 432743.10386. Following computational analysis, *in vitro* validation demonstrated that Peptide C and Peptide E effectively induced apoptosis in SW620, SW480, and HCT116 colorectal cancer cell lines while sparing normal human colon epithelial cells (CCD841) (Hashem et al., 2025). This selective pro-apoptotic activity underscores their potential as targeted anticancer agents with minimal off-target toxicity. Nevertheless, further investigation, particularly *in vivo* organ toxicity assessments, is required to confirm their safety and support their clinical translation. To address this, we utilized the zebrafish model, renowned for its genetic and physiological similarity to humans. Comprehensive preclinical evaluations and further validation are imperative to confirm the safety and efficacy of these peptides *in vivo*, enabling confident progression to subsequent developmental stages and potentially introducing novel and effective treatments for colorectal cancer.

This study specifically investigated the *in vivo* organ-specific safety profiles (cardiac, neuromuscular, and hepatic) of Peptide C and Peptide E in zebrafish, aiming to elucidate potential human and environmental health risks.

## 2 Material and methods

### 2.1 Peptide design

In this study, we used two previously characterized bioinspired short antimicrobial peptides (BSAMPs): Peptide C (GVLCCGYRCCSKWGWCGTT) and Peptide E (CWWMTRRAWR). These peptides were previously shown to exert selective anticancer and pro-apoptotic effects *in vitro* against SW620, SW480, and HCT116 colorectal cancer cell lines, without inducing cytotoxicity in the normal colon epithelial cell line CCD841 (Hashem et al., 2025). Before proceeding to *in vivo* evaluation, we assessed their predicted cytotoxicity, immunogenicity, and blood half-life through *in silico* analysis. The cytotoxic potential of both BSAMPs was evaluated using the ToxiPred online tool [https://webs.iiitd.edu.in/raghava/toxinpred/]. Peptide C exhibited moderate hydrophobicity (26%) and moderate basicity (10%), corresponding to a predicted cytotoxicity probability of approximately 0.30, suggesting a low to moderate risk of cytotoxicity. These properties support selective anticancer activity while minimizing toxicity toward normal cells. In contrast, Peptide E demonstrated high hydrophobicity (50%) and high basicity (30%), resulting in a predicted cytotoxicity probability of approximately 0.75, indicative of a high likelihood of cytotoxicity. However, selective targeting of cancer cells remains possible due to differences in membrane composition. Table 1 summarizes the cytotoxicity profiles of Peptide C and Peptide E.

**TABLE 1:** Predicted cytotoxicity characteristics of BSAMPs.

Peptide sequence	Name	Length (aa)	Hydrophobic content (%)	Basic residue content (%)	Predicted cytotoxicity probability	Predicted cytotoxicity level
GVLCCGYRCCSKWGWCGTT	Peptide C	19	26.3	10.5	0.30	Low to Moderate
CWWMTRRAWR	Peptide E	10	50.0	30.0	0.75	High

To evaluate the immunogenicity of Peptides C and E, we used the Immune Epitope Database (IEDB) [https://www.iedb.org], screening based on common colon cancer antigenic patterns. For Peptide C, its moderate length, presence of cysteine residues (which can aid MHC binding while requiring proper folding), and the absence of strong polybasic or polyaromatic motifs typically associated with immunodominant epitopes suggest a low to moderate immunogenic potential. For Peptide E, the peptide’s length (10 amino acids) is optimal for MHC class I presentation, and the clustering of basic residues may enhance MHC stabilization but could slightly reduce solubility. Basic sequence screening revealed no significant matches to known strong human T-cell epitopes in the IEDB database. Thus, Peptide E is predicted to have low to moderate immunogenicity, slightly higher than Peptide C, due to its favorable length for MHC-I binding. Table 2 summarizes the immunogenicity characteristics of both BSAMPs.

**TABLE 2:** Predicted immunogenicity characteristics of BSAMPs.

Peptide sequence	Length (aa)	MHC class I binding affinity (IC50 nM)	Immunogenicity score	Interpretation
GVLCCGYRCCSKWGWCGTT	19	250	0.15	Low
CWWMTRRAWR	10	50	0.35	Moderate

Furthermore, the *in silico* evaluation of half-life and blood stability was conducted using PlifePred [https://webs.iiitd.edu.in/raghava/plifepred/]. Peptide C exhibited a predicted half-life of 954.61 s, while Peptide E demonstrated a half-life of 840.31 s, indicating a favorable stability profile for potential therapeutic applications. The molecular mass of the peptides was calculated using the Peptide Molecular Weight Calculator [https://www.peptide2.com/peptide_molecular_weight_calculator.php], resulting in a molecular mass of 2,211.67 Da for Peptide C and 1,451.75 Da for Peptide E. Both peptides were synthesized by NovoPro Labs [https://www.novoprolabs.com/custom-peptide-synthesis/], a specialized custom peptide synthesis provider.

### 2.2 Peptides antimicrobial activity information

Peptide C demonstrated antimicrobial activity against several bacterial strains, including *Staphylococcus aureus* (128 μg/mL), *Pseudomonas aeruginosa* (1,024 μg/mL), and *Acinetobacter baumannii* (512 μg/mL). In addition, Peptide C exhibited *in vitro* antifungal activity against pathogenic fungi, including *Candida albicans* (128 μg/mL), *Candida krusei* (256 μg/mL), *Candida parapsilosis* (1,024 μg/mL), and *Candida glabrata* (1,024 μg/mL) (unpublished data; protected under Intellectual Property disclosure #QU 2024-009).

### 2.3 Chemicals

Zinc oxide (ZnO) dispersion (nanoparticles) of diameter <100 nm from Sigma-Aldrich (St. Louis, MO, United States) was used as a positive control, as it causes morphological deformities in the embryos of zebrafish and is also known to cause mortality. Other toxicological studies also use it as a positive control (Al-Jamal et al., 2020; Al-Kandari et al., 2019). The zebrafish embryos were grown in N-Phenylthiourea (≥98% PTU) (Sigma-Aldrich, United States) *in vitro*, as it inhibits the formation of pigment in the embryo and thus helps with uncomplicated visualization of the embryos under the microscope (Nasrallah et al., 2018). PTU was used as the negative control and was prepared in a 60X stock of egg water solution and a 60X stock of PTU solution dissolved in up to 5 L of MilliQ water. Embryos were also incubated in E3 (egg water) media (Sigma-Aldrich, Steinheim, Germany) to grow efficiently with pigmentation and used as a negative control. The components of E3 media consist of 5.0 mM NaCl, 0.17 mM KCl, 0.16 mM MgSO4 ⋅ 7H2O, and 0.4 mM CaCl_2_ ⋅ 2H_2_O – all materials from Sigma-Aldrich (Steinheim, Germany) prepared in 1 L of MilliQ water. MilliQ water is water purified using the MilliQ water purification system. Pronase enzyme (Sigma, Steinheim, Germany) was used to dechorionate the embryos, as the chorion around the embryo would interfere with the peptide uptake for the treatment. Tricaine-S (Syndel, Washington, United States) was used as an anesthetizer for imaging the larvae, and Methylcellulose (Sigma-Aldrich, Germany) was used for positioning the larvae laterally or ventrally for imaging. The stock solutions of Peptide C (NovoPro-peptide 23162-2 Lot# GTA33702-2-0909, Shanghai, China) of sequence GVLCCGYRCCSKWGWCGTT and Peptide E (NovoPro- peptide 23162-1 Lot# GTA33702-1-0909, Shanghai, China) of sequence CWWMTRRAWR of concentration 50 mg/mL was prepared by measuring 0.05 g of powdered stock in 1 mL of MilliQ water and mixing through vortex to check the effects of its toxicity.

### 2.4 Zebrafish housing and breeding

The zebrafish embryos of various transgenic reporter lines (myl7: eGFP, olig2: dsRed, and Fabp: 10 dsRed) and wild type were used to assess the toxicity. The zebrafish were maintained in an environmentally controlled lab (photoperiod 14 h light/10 h dark cycle and water temperature of 28°C) with filtering systems at the zebrafish core facility in Sidra Medicine, Qatar. One day before the mating, the male and female fish were separated in the breeding tanks using the divider. The spawning was triggered the next day by removing the divider, which allowed the zebrafish to mate. The embryos were collected and washed with PTU or E3 media and incubated in PTU (with Methylene blue) or E3 media in a petri dish for 24 h inside an incubator maintained at a temperature between 27°C and 28°C (Al-Jamal et al., 2020). The organs of the zebrafish develop and become functional at different stages. The cardiotoxicity and motor neurons were measured at 72 hpf, whereas the locomotion and liver toxicity were measured at 120 hpf. In this research study, all the different experiments were according to the Ministry of Public Health Guidelines assigned by the Institutional Animal Care and Use Committee (IACUC) committee - Qatar Foundation, Doha – Qatar citing the usage of embryos beyond five dpf as unethical and immediate ethnicization of the embryos beyond 5dpf (MoPH-Policy-on-Zebrafish-Research.pdf (evmc.qa).

### 2.5 Developmental (acute toxicity) assay

The toxicity levels of BSAMPS Peptide C (GVLCCGYRCCSKWGWCGTT) and Peptide E (CWWMTRRAWR) were measured by mortality and acute toxicity assay. Zebrafish embryos of 24 hpf were separated from severely delayed and dead embryos, which were then dechorionated using 0.8 mL of 10 mg/mL of pronase with five times extensive washing in 6-well plates that contained 35 embryos in each well. The healthy dechorionated embryos were then incubated in 5 mL of i) Peptide C concentrations (10, 50, 100, 150, 200, 500, 750 and 1,000 μg/mL), ii) Peptide E concentrations (10, 25, 50, 75, 100, 150, 200, 400, 600 and 1,000 μg/mL), iii) positive control – ZnO (30 mg/L) and iv) negative control – PTU. The mortality and morphological deformation for the treated groups were assessed over three consecutive days (48, 72, and 96 hpf) under a standard stereoscopic microscope (Al-Jamal et al., 2020). Deformities such as scoliosis, yolk sac, pericardial edema, or pigmentation were observed over the 3 days by gross microscopic examination, and the embryos were then grouped into three groups for classification (G1 – severely deformed, G2 – moderately deformed, G3 – normal). The mortality percentage was calculated by counting the number of dead embryos over the total number of embryos used for each treatment. The lethal concentration (LC50) was calculated by plotting a sigmoidal curve to mortality data, and the concentrations for organ-toxicity assays were assessed through mortality percentage (Al-Jamal et al., 2020). Group images for larvae at 96 hpf of different groups were imaged using an Axio Zoom stereomicroscope, Zeiss (Germany), with a magnification of 12.5x using Zeiss AxioCam ERc 5 with embryos in 2.5% methylcellulose for positioning imaging.

### 2.6 Cardiovascular toxicity

The zebrafish’s transgenic cardiac myosin light chain 2 gene-GFP Tg (myl7:eGFP) strain was used to perform the cardiotoxicity assays (Abou-Saleh et al., 2019; Al-Jamal et al., 2020). The assessment involved the blood flow rate and heartbeat of 72 h post-fertilized embryos. The 24 hpf (Peptide C) and 48 hpf (Peptide E) healthy embryos were dechorionated by treating them with 0.8 mL of pronase enzyme with extensive five times washing in PTU solution. The embryos were then transferred into 6–well plates for incubation in 5 mL of i) Peptide C (100, 150, and 200 μg/mL), ii) Peptide E (10, 30, and 60 μg/mL), iii) positive control – ZnO (30 mg/L) and iv) negative control (PTU). The incubation period for dosage effects for Peptide C was 2 days (24–72 h), and for Peptide E, it was 1 day (48–72 h). At 72 hpf, the embryos were anesthetized using Tricaine (4 mg/mL) and kept on the depression slide with 1 to 2 drops of 2.5% methylcellulose (Sigma-Aldrich, United States) positioned ventrally for imaging. Using the Axio Zoom stereomicroscope, Zeiss (Germany), the blood flow rate and heart rate were recorded at 60 frames per second (fps) using Imaging Source camera (Germany) at 112x magnification. Then, the videos were converted from TIF to AVI format using ImageJ open software, and the analysis was done manually using Danioscope analysis software from Noldus Technology (Netherlands). The analysis was used to track i) the blood flow rate percentage of the dorsal aorta (DA) and posterior cardinal vein (PCV), and ii) the beats per minute of the ventricle and atrium of the heart.

### 2.7 qRT-PCR analysis of cardiac stress marker gene expression

Total RNA was extracted from zebrafish samples, and RT-PCR was performed to express key cardiac failure markers Atrial Natriuretic Factor (ANF)/Natriuretic Peptide Precursor A (NPPA). NPPA is a gene in the heart that encodes for ANF. The cDNA synthesis was then carried out using the Prime Script^®^ RT reagent kit, with ≈1 μg of total RNA. The relative quantification was normalized to β-actin (housekeeping gene), which served as an internal control. The results obtained were presented as 2^−ΔΔCT^. The primer sequence for NPPA was GCT​CCT​GGT​TTG​GCA​GCA​G (Forward Primer) and GAG​CTG​CTG​CTT​CCT​CTC​GG (Reverse Primer) (Wei et al., 2021).

### 2.8 Neurotoxicity assay

The transgenic Tg (oligo2: dsRed) strain was crossed with wild-type AB zebrafish. The oligo strain of zebrafish is used to perform neurology assays as it has fluorescence markers for all motor neurons (Al-Jamal et al., 2020; Pagnamenta et al., 2021).

#### 2.8.1 Axon imaging

The embryos were collected and incubated in PTU for ease of imaging. The healthy embryos at 24 hpf were screened for positive embryos with fluorescent brain and spinal cord. The positive embryos were then dechorionated with 0.8 mL pronase and extensive washing. The healthy dechorionated embryos were transferred to the six-well plate and incubated in 5 mL of i) Peptide C (100, 150, and 200 μg/mL), ii) Peptide E (10, 30, and 60 μg/mL), iii) positive control–ZnO (30 mg/L) and iv) negative control (PTU). The incubation period for Peptide C was 2 days (24–72 h), and for Peptide E, it was 1 day (48–72 h). At 72 hpf, the embryos were imaged for axons using Vast BioImager (UnionBiometrica, Spain) in 20x magnification after anesthetizing with Tricaine (4 mg/mL). The red images were converted to black using Fiji ImageJ open-source software. Danioscope analysis software from Noldus Technology (Netherlands) was used to measure the axon length from the 6th to the 14th.

#### 2.8.2 Locomotion assay

The embryos were collected and incubated in egg water for ease of detection. The healthy embryos at 24 hpf were collected and dechorionated with 0.8 mL pronase and extensive washing. The healthy dechorionated embryos were transferred to the six-well plate and incubated in 5 mL of i) Peptide C (100, 150, and 200 μg/mL), ii) Peptide E (10, 30, and 60 μg/mL), iii) positive control – ZnO (30 mg/L) and iv) negative control (PTU). The incubation period for Peptide C was 4 days (24–120 h), and for Peptide E, it was 1 day (96–120 h). At 120 hpf, the movement of the embryos was measured using Ethovision XT software (Noldus Technology, Netherlands). The movements were measured under the following conditions: an initial 2 min of darkness accompanied by 2 min of repeated bright light cycles separated by 2 min of darkness for 12 min. The analysis was followed by measuring the average total distance moved, average velocity, and rotation of the embryos.

### 2.9 Hepatotoxicity

Tg (Fab10p: dsRed) transgenic strain of zebrafish was used to perform the hepatotoxicity assay, as this strain expresses red fluorescent protein (RFP) in the hepatocytes of the zebrafish, thus allowing good-quality imaging of the liver (Abou-Saleh et al., 2019; Al-Jamal et al., 2020; Al-Asmakh et al., 2020). The assessment involved measuring the area of the liver of 120 h of fertilized embryos. The 24 hpf (Peptide C) and 96 hpf (Peptide E) healthy embryos were dechorionated by treating them with 0.8 mL pronase with extensive 5 times washing in PTU solution. The embryos were then transferred into 6–well plates for incubation in 5 mL of i) Peptide C (100, 150, and 200 μg/mL), ii) Peptide E (10, 30, and 60 μg/mL), iii) positive control – ZnO (30 mg/L) and iv) negative control (PTU). The incubation period for dosage effects for Peptide C was 4 days (24–120 h), and for Peptide E, it was 1 day (96–120 h). The fluorescent red liver was imaged using Vast Bioimager (UnionBiometrica, Spain) at 20x magnification, and the analysis of the liver size was done manually using the DanioScope software (Noldus Technology, Netherlands) after converting it into black images using Fiji ImageJ open-source software.

### 2.10 Statistical analysis

Descriptive statistics are presented as mean ± standard error of the mean (SEM) for cardiotoxicity, neurotoxicity, hepatotoxicity, and locomotion assays. Bar graphs were used to visualize the data. Given the presence of unequal variances and sample sizes across treatment groups, Welch’s t-test was employed for group comparisons, as it offers more robust control of Type I error under these conditions. This test was chosen over ANOVA due to the lack of variance homogeneity. Significant outliers were identified and removed using GraphPad Prism 9 software. Statistical significance was considered at *p < 0.05, **p < 0.01, ***p < 0.001, and ****p < 0.0001.

## 3 Results

### 3.1 Peptide C and peptide E exhibit no acute toxicity at low concentrations on zebrafish embryos

The experiments were conducted in a controlled laboratory setting where the environmental conditions of the water were regulated. The adult zebrafish were housed in a circulating housing system with controlled temperature, pH, and salinity set points and an automated dosing system. At the same time, the embryos were specifically maintained at a fixed temperature of 28.5°C in an incubator. This controlled setup ensured that any observed effects could be attributed to the variables of peptide treatment being studied rather than uncontrolled environmental factors.

The acute toxicity effects of peptides on zebrafish development were first measured by determining the survival rate of zebrafish embryos treated with peptides C and E. Zebrafish embryos were collected and dechorionated after 24 h post-fertilization (hpf). Dechorionated embryos were then treated in (i) various concentrations of Peptide C (10, 50, 100, 150, 200, 500, 750, and 1,000 μg/mL), (ii) various concentrations of Peptide E (10, 25, 50, 75, 100, 150, 200, 400, 600, and 1,000 μg/mL), (iii) N-Phenylthiourea (PTU) (negative control), and (iii) Zinc Oxide (ZnO; 30 μg/mL) (positive control). The embryos were observed at 48 hpf, 72 hpf, and 96 hpf (Al-Jamal et al., 2020). To assess the toxicity of peptides on zebrafish embryos, the LC50 was determined by calculating the mortality rates of embryos from 24 hpf to 96 hpf. For Peptide C, the highest concentration, which does not cause any significant difference when compared to the control group, was 200 μg/mL (No Observed Effect Concentration). No observable impact on embryonic development or gross morphological traits, including scoliosis, body length variations, and yolk sac morphology, was detected following the incubation of zebrafish embryos with Peptide C at a 200 μg/mL concentration for 4 days. For Peptide E, the NOEC could not be calculated as it showed cumulative toxicity, and thus, all the experiments were treated for 1 day. Given the observed differences in toxicity profiles, different exposure durations were selected for Peptide C and Peptide E. Peptide C exhibited gradual, concentration-dependent toxicity without immediate lethality, permitting a 4-day exposure to evaluate both acute and subtle developmental effects. In contrast, Peptide E showed cumulative and rapid toxicity at concentrations above 100 μg/mL, leading to complete embryonic mortality before 96 h post-fertilization. Therefore, a 24-h exposure was chosen for Peptide E in subsequent organ-specific toxicity assessments to capture early sublethal effects without interference from systemic lethality.

**FIGURE 1:**
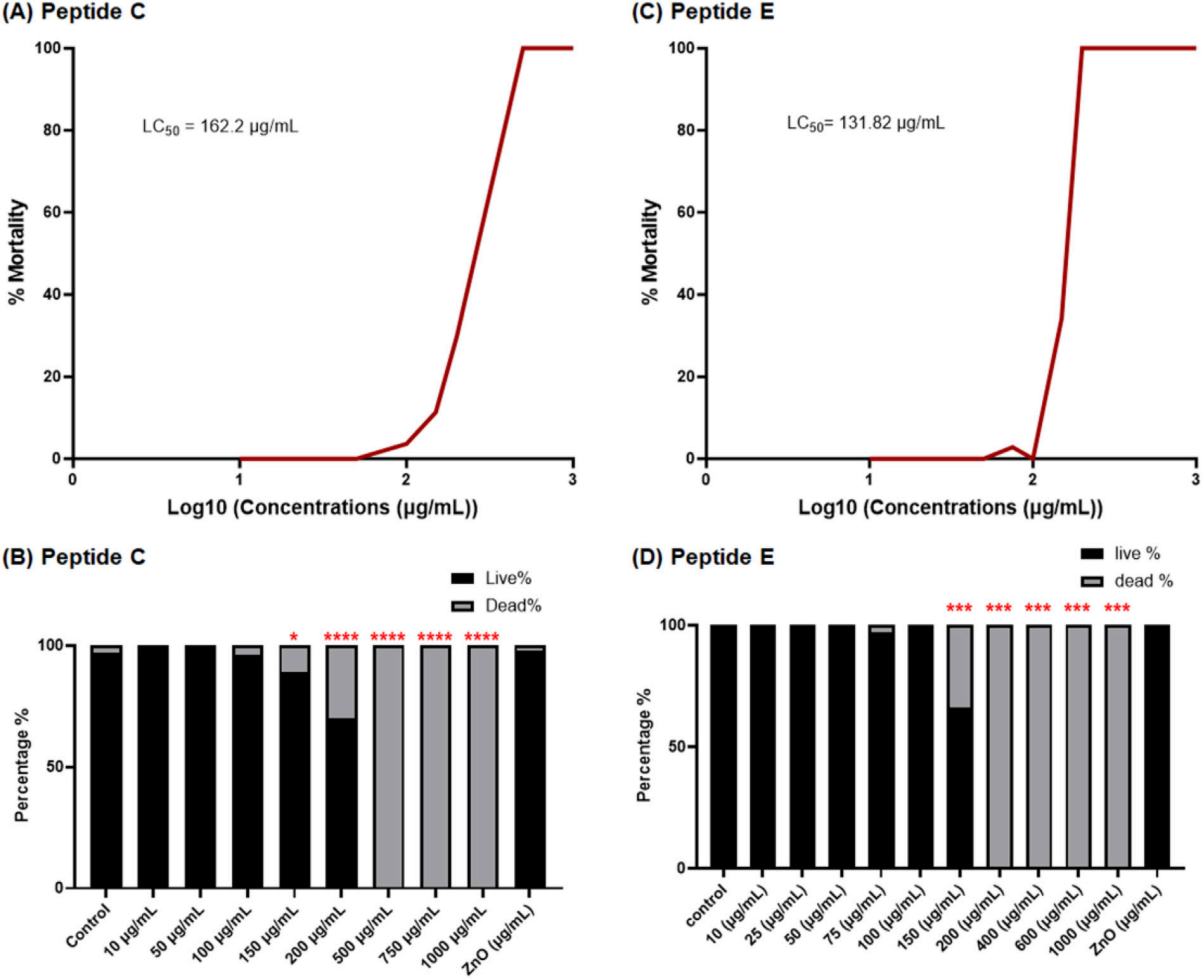
Viability of zebrafish embryos exposed to different concentrations of Peptide C and Peptide E. **(A,C)** LC50 concentration–response curves for Peptide C and Peptide E, respectively, based on zebrafish embryo viability. **(B,D)** Percentage of cumulative survival rates of zebrafish embryos treated with different concentrations of Peptide C and Peptide E, respectively, monitored until 96 h post-fertilization (hpf). Statistical analysis was performed using Welch’s t-test to compare each treatment group to the negative control. Statistical significance: *p < 0.05, **p < 0.01, ***p < 0.001, ****p < 0.0001 compared to the control.

The survival rate of embryos reveals a concentration-dependent decrease, indicating a concentration-dependent effect of Peptide C (Figure 1A). Interestingly, no gross morphological abnormalities were observed in the embryos. The survival percentage was 100% for embryos treated with 10 and 50 μg/mL of Peptide C. However, the survival percentage decreased to 97% for embryos treated with 100 μg/mL, 87% for the treatment with 150 μg/mL, and 64% for the treatment with 200 μg/mL. At higher concentrations of 500, 750, and 1,000 μg/mL, mortality was observed in 100% of treated embryos (Figure 1B). The cumulative survival percentage was recorded at 96 hpf and was used to construct the sigmoidal curve to identify the LC50 values of the peptide. The peptide C LC50 value was found to be at 162.2 μg/mL (Figure 1A). These results suggest that Peptide C exhibits a concentration-dependent toxicity effect on zebrafish embryos. The LC50 value for Peptide E was 131.82 μg/mL (Figure 1C). The concentrations starting from 10 μg/mL to 100 μg/mL were non-toxic to the embryos, with 100% survival, and mild toxicity was observed at 150 μg/mL, with 71.87% survival. However, concentrations beyond 150 μg/mL exhibited global toxicity with 100% mortality of the embryos (Figure 1D).

The prolonged exposure of zebrafish embryos to Peptide E at concentrations higher than 100 μg/mL is highly toxic and leads to 100% mortality by 96 hpf (Figure 1D). Also, the cumulative toxicity of Peptide E within the embryos is evident. Therefore, to assess the organ-specific safety profile, the concentrations of Peptide E were lowered to concentrations of 10, 30, and 60 μg/mL treatment for 24 h to examine and assess the effect on the selected organ system.

### 3.2 Cardiovascular assay

**FIGURE 2:**
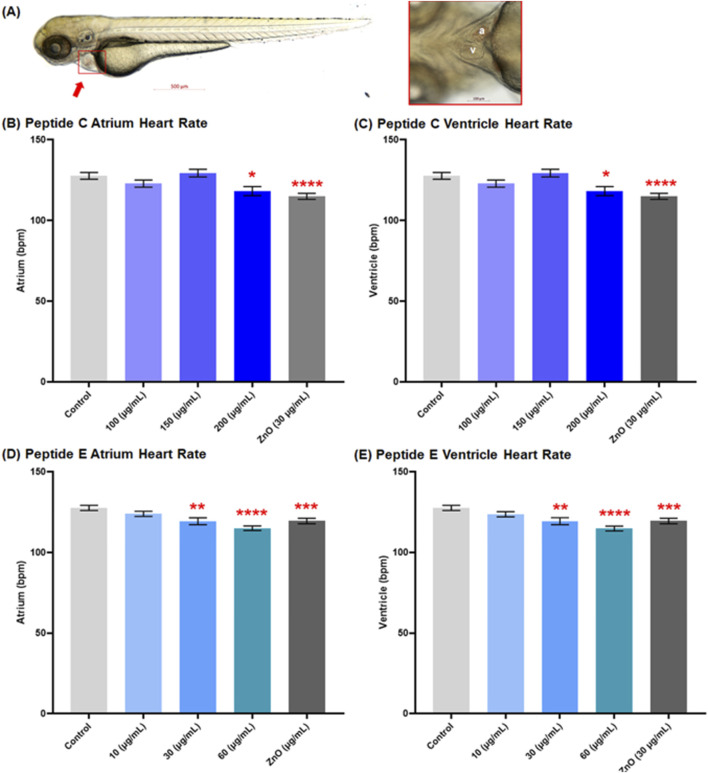
Effect of Peptides C and E on the heart rate of zebrafish embryos. **(A)** Representative image of a control zebrafish embryo at 72 h post-fertilization (hpf), captured using an Axio Zoom stereomicroscope (Zeiss, Germany). The left panel shows a lateral view of the full body with the heart indicated by a red arrow; the right panel shows a ventral view of the heart (a: atrium, v: ventricle). Heart rates (beats per minute, bpm) in the atrium and ventricle were measured using Danioscope software (Noldus Technology, Netherlands) after video acquisition at 60 frames per second and 112× magnification. Graphs show the heart rate effects of Peptide C in the **(B)** atrium and **(C)** ventricle, and Peptide E in the **(D)** atrium and **(E)** ventricle. Data represent the mean ± standard error of the mean (SEM). Statistical analysis was performed using Welch’s t-test to compare each treatment group to the negative control. Statistical significance: *p < 0.05, **p < 0.01, ***p < 0.001, ****p < 0.0001 compared to the negative control.

**FIGURE 3:**
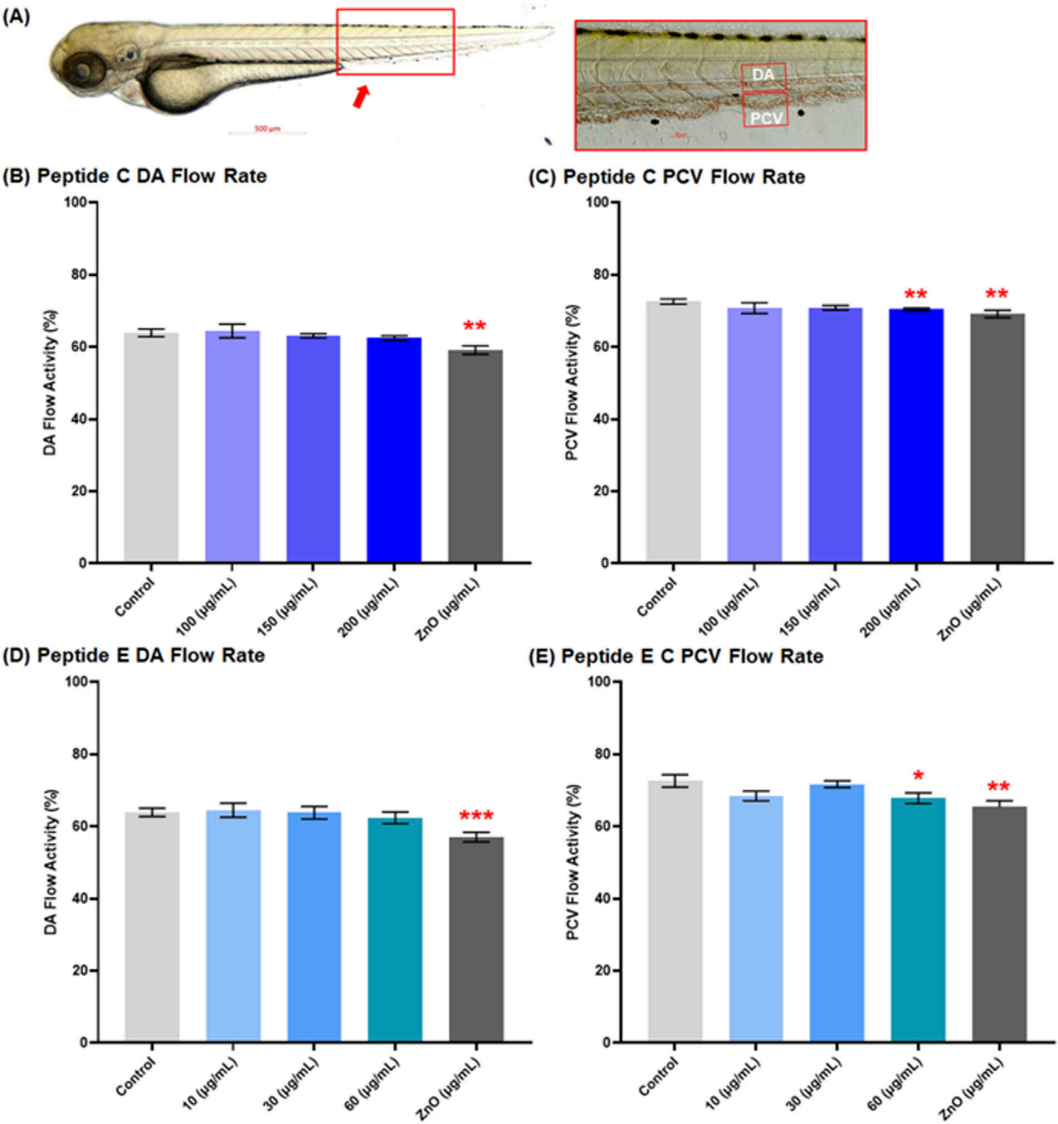
Effect of Peptides C and E on the blood flow of zebrafish embryos. **(A)** Representative image of a control zebrafish embryo at 72 h post-fertilization (hpf), captured using an Axio Zoom stereomicroscope (Zeiss, Germany). The red box indicates the dorsal region used for assessing vascular activity. The inset shows the lateral view of the main vasculature: dorsal aorta (DA) and posterior cardinal vein (PCV). Zebrafish blood flow was recorded at 60 frames per second using an Imaging Source camera (Germany) at ×112 magnification. Blood flow rates in the DA and PCV were analyzed using Danioscope software (Noldus Technology, Netherlands). A total of 15 embryos were analyzed per treatment group. Graphs illustrate the effect of Peptide C on **(B)** DA and **(C)** PCV flow rate, and Peptide E on **(D)** DA and **(E)** PCV flow rate. Values are expressed as mean ± standard error of the mean (SEM). Statistical analysis was performed using Welch’s t-test to compare each treatment group to the negative control. Statistical significance: *p < 0.05, **p < 0.01, ***p < 0.001, and ****p < 0.0001 compared to the negative control.

In this experiment, we employed the transgenic zebrafish line (myl7: eGFP: expressing GFP in cardiomyocytes) to assess the cardiac safety profiles of Peptides. Zebrafish embryos were collected and subsequently exposed to various concentrations of Peptide C (100, 150, and 200 μg/mL), Peptide E (10, 30, and 60 μg/mL), as well as PTU (negative control) and ZnO (30 μg/mL, positive control). The 72 hpf embryo possesses nearly all cardiac components suitable for analyzing cardiotoxicity (Al-Ansari et al., 2022). Therefore, at 72 hpf, the main vasculature and heart were examined. The heart rate (atrium and ventricle, Figure 2) and the blood flow of the dorsal aorta and post-cardinal vein (Figure 3) were recorded and imaged.

### 3.3 Peptide C and peptide E decrease the heart rate at moderate concentrations

Figure 2A shows a representative image of a control zebrafish embryo at 72 hpf. The left panel displays a lateral view of the full body, with the heart indicated by a red arrow; the right panel highlights a ventral view of the heart, clearly showing the atrium (a) and ventricle (v). These anatomical references were used to guide imaging and quantification of cardiac parameters. For Peptide C, the results demonstrated a decrease in heart rate as concentrations increased compared to the control, establishing a concentration-dependent effect of peptide C on heart function. The mean heart rate values for control, 100, 150, and ZnO µg/mL were 127.7 bpm ±2.10, 122.9 bpm ± 2.19, 129.4 bpm ± 2.36, 118.2 bpm ± 2.81, and 115.0 bpm ±1.90, respectively (Figures 2B,C).

To assess the cardiotoxicity potential of Peptide E, the beats per minute within the atrium and ventricle of zebrafish embryos were calculated after a 24-h treatment in different concentrations, starting at 48 hpf to 72 hpf. The incubation with Peptide E indicated a concentration-dependent decrease in heart rate, with a statistically significant reduction at the higher concentration of 30 and 60 μg/mL compared to the control (Figures 2D,E). There was a significant decrease at 60 μg/mL (115.1 ± 1.38; 114.8 ± 1.50) bpm and 30 μg/mL (119.4 ± 2.17; 119.4 ± 2.17) bpm, with no effect at 10 μg/mL (124.0 ± 1.59; 123.7 ± 1.58) bpm when compared to the control (PTU) (127.7 ± 1.60; 127.7 ± 1.60) bpm for atrium and ventricle, respectively. All groups across the atrium and ventricle show similar heart rates except for the concentrations of 10 and 60 μg/mL, suggesting heart arrhythmia (Zhu et al., 2014).

### 3.4 Peptides C and peptide E decrease blood flow at high concentrations

The impact of peptides on the cardiac system’s function focused on the blood flow rate percentage in the dorsal aorta (DA) and the post-cardinal vein (PCV), the main vasculature. Representative imaging of control embryos at 72 hpf is shown in Figure 3A, with the red box highlighting the dorsal region used for vascular flow assessment. For Peptide C, the results demonstrated a decrease in the blood flow rate percentage in both vessels in a concentration-dependent trend. The mean values for the control, 100, 150, 200, and ZnO 30 μg/mL for the DA were 63.9% ± 1.07, 64.45% ± 1.89, 63.1% ± 0.60, 62.49% ± 0.61, and 59.14% ± 1.178, respectively (Figure 3B); and for the PCV were 72.61% ± 0.72, 70.77% ± 1.49, 70.85% ± 0.64, 70.41% ± 0.35, and 69.13% ± 1.04, respectively (Figure 3C).

For Peptide E, the blood flow rate percentage was calculated after a 1-day treatment of zebrafish embryos at different concentrations, starting at 48 hpf and concluding at 72 hpf. There was an observed decrease in the blood flow rate of DA at 10 μg/mL (64.5% ± 1.94), 30 μg/mL (63.78% ± 1.73), 60 μg/mL (62.39% ± 1.59), and ZnO (30 μg/mL) (57.02% ± 1.33) compared to the control DA flow rate of 63.90% ± 1.16 (Figure 3D); and for the PCV flow rate was 10 μg/mL (68.44% ± 1.35), 30 μg/mL (71.70% ± 0.92), 60 μg/mL (67.82% ± 1.47) and ZnO 30 μg/mL (65.37 ± 1.75) compared to the control PCV flow rate of 72.61% ± 1.73 (Figure 3E). However, the overall trend of various treatment effects on blood flow rate did not indicate any significant changes at low to moderate concentrations.

### 3.5 Peptides C and peptide E induce cardiac stress by increasing ANP/NPPA expression

**FIGURE 4:**
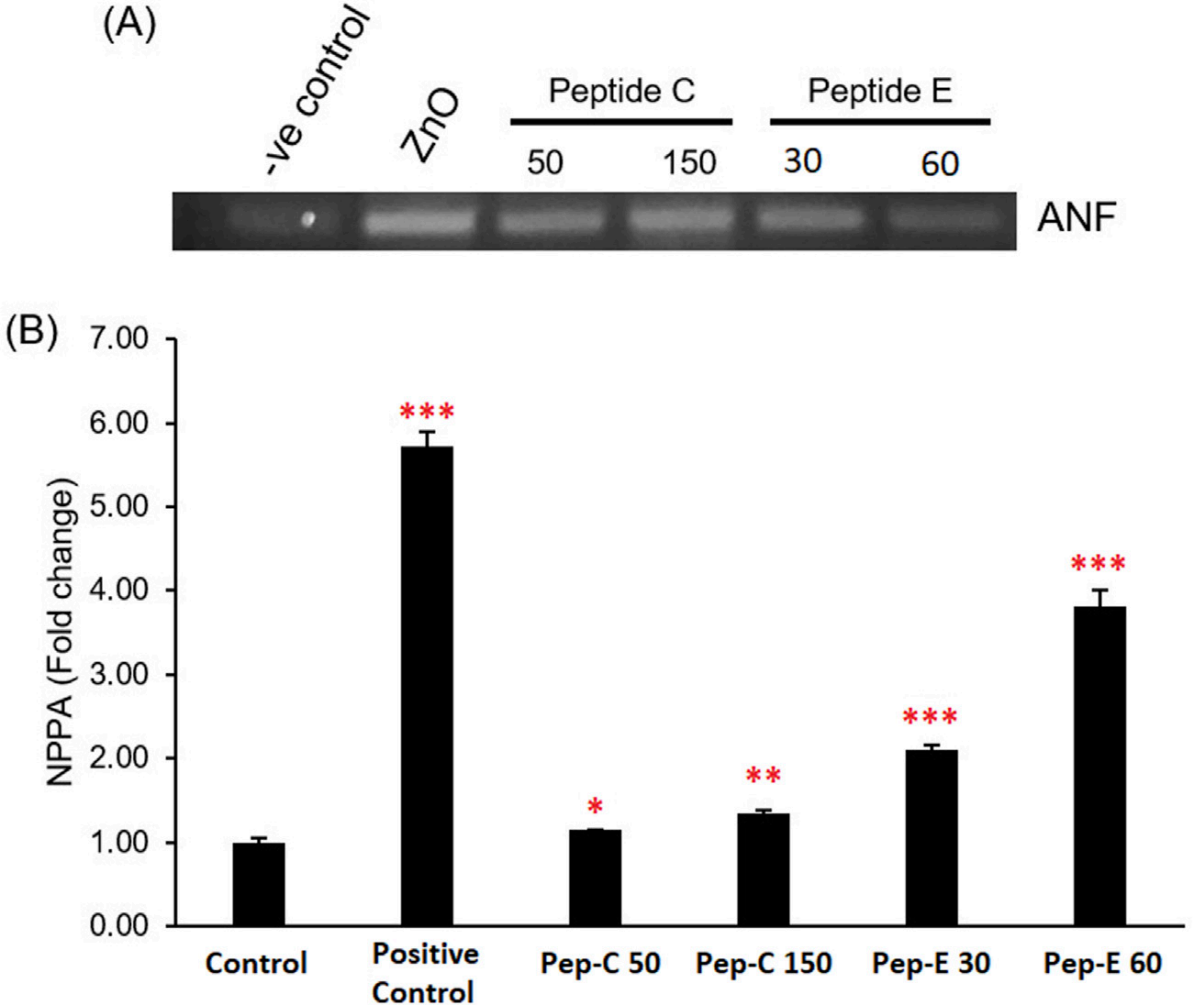
Effect of Peptides C and E on the expression of the cardiac stress marker ANF/NPPA in zebrafish embryos. **(A)** Representative RT-PCR gel image showing atrial natriuretic factor (ANF/NPPA) expression levels following treatment with Peptide C (50 and 150 μg/mL), Peptide E (30 and 60 μg/mL), and ZnO (30 μg/mL, positive control). **(B)** Bar graph representing NPPA fold change relative to the untreated control group. Expression was quantified using densitometry and normalized to the control. Each treatment was performed in quadruplicate (n = 4 per group). Data are presented as mean ± SEM. Statistical analysis was performed using Welch’s t-test to compare each treatment group to the negative control. Statistical significance: *p < 0.05, **p < 0.01, and ***p < 0.001 compared to the negative control.

ANP, or atrial natriuretic peptide, serves as a cardiac hormone crucial for regulating blood pressure and fluid balance. In studies focused on cardiotoxicity, alterations in ANP expression levels can serve as indicators of cardiac stress or damage. To evaluate the cardiotoxic effects of the peptides on zebrafish, we analyzed changes in ANP expression, specifically assessing ANF/NPPA expression levels (Wei et al., 2021). RT-PCR was performed on the 96 hpf zebrafish embryos. Zebrafish embryos were collected and treated with 50 and 150 μg/mL of Peptide C and Peptide E. It was established that the treatments of Peptide C and E led to a significant increase in the expression of ANP/NPPA (Figure 4).

This increase in marker expression confirmed that both peptides induce cardiac stress in the zebrafish and could hinder the functional maturation of the zebrafish heart, leading to a decline in the heart rate and reduced blood flow rate percentage.

### 3.6 Neurological assay

#### 3.6.1 Peptide C but not peptide E shortens motor axon

**FIGURE 5:**
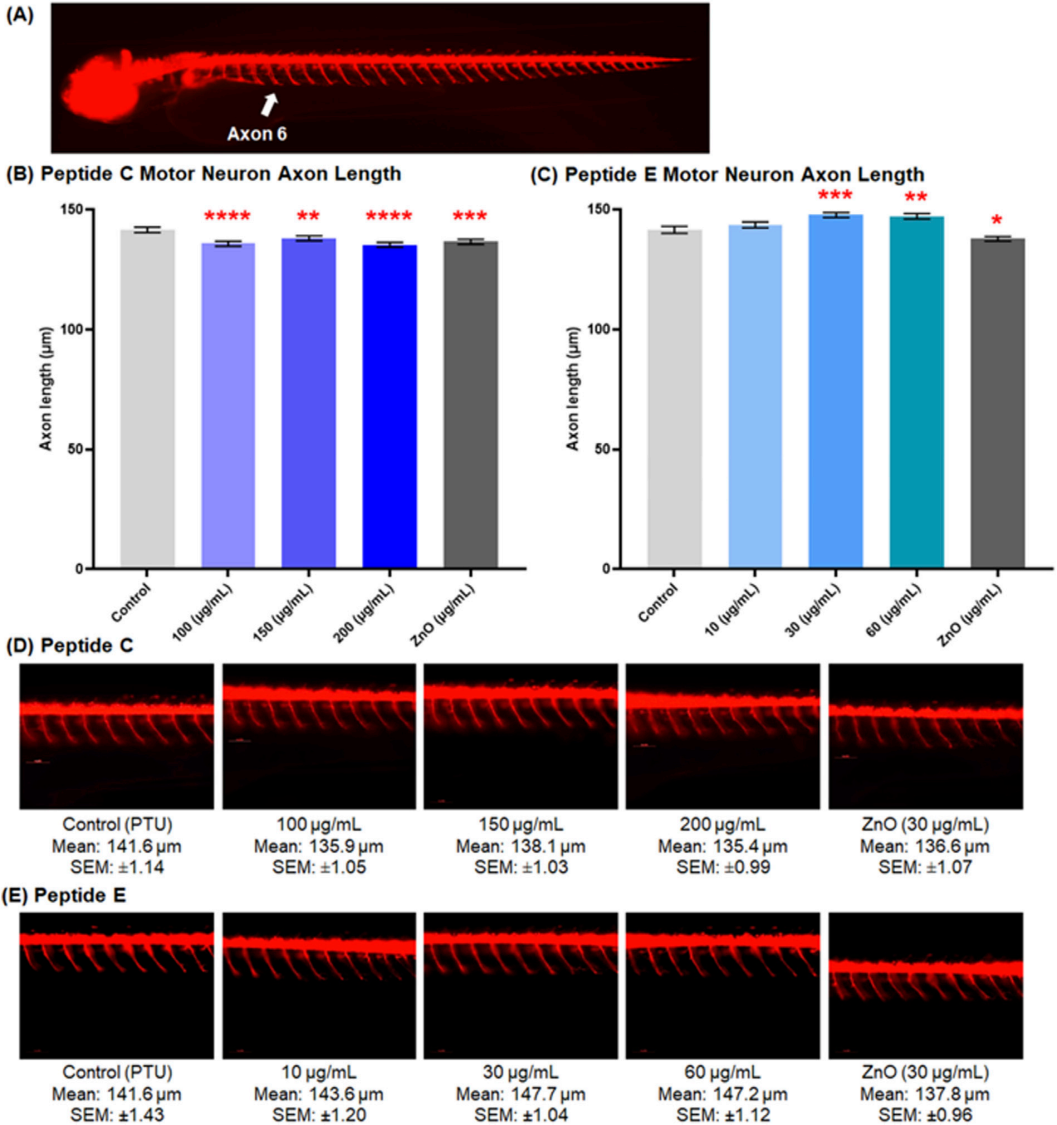
Peptide C and E effects on spinal motor neuron axon length in 72 hpf zebrafish embryos. **(A)** Representative image showing motor neuron axons spanning from axon 6 to 14 (5× magnification). Zebrafish embryos were treated with different concentrations of Peptide C and Peptide E. Images of the tail region showing fluorescent motor neurons innervating muscle were captured at 20× magnification using the VAST BioImager (Union Biometrica, Spain). A total of 12 embryos (Peptide C) and 11 embryos (Peptide E) were analyzed per treatment group. Axon lengths were measured using DanioScope software (Noldus Technology, Netherlands). **(B)** Quantitative analysis of axon length in embryos treated with Peptide C. **(C)** Quantitative analysis of axon length in embryos treated with Peptide E. **(D, E)** Representative images of motor neuron projections from embryos treated with various concentrations of Peptide C **(D)** and Peptide E **(E)**. Data are presented as mean ± SEM. Statistical analysis was performed using Welch’s t-test to compare each treatment group to the negative control. *p < 0.05, **p < 0.01, ***p < 0.001, and ****p < 0.0001 as compared to the negative control.

The neurotoxicity assay is assessed in the transgenic zebrafish line (oligo2: dsRed) strain (Al-Jamal et al., 2020; Pagnamenta et al., 2021). Zebrafish embryos were collected and treated with different concentrations of Peptide C, Peptide E, PTU (negative control), and ZnO (positive control). Figure 5A shows a representative image of motor neuron axons extending from somites 6 to 14. Axonal morphology, length, and integrity were qualitatively and quantitatively assessed.

Treatment with Peptide C resulted in a significantly shorter motor axon length compared to the negative control. The mean values for the control, 100, 150, 200, and ZnO 30 μg/mL of the axons were 141.6 μm ± 1.14, 135.9 μm ± 1.05, 138.1 μm ± 1.03, 135.4 μm ± 0.99, and 136 μm ± 1.07, respectively (Figure 5B). For Peptide E, the axonal length showed a different effect. There was no effect in the 10 μg/mL (143.6 μm ± 1.20) treated zebrafish group compared to the control axon length (141.6 μm ± 1.43). However, the motor neuron axon length was significantly longer at 30 μg/mL (147.7 μm ± 1.04), followed by 60 μg/mL (147.2 μm ± 1.12) treatments (Figure 5C). Representative images of motor neuron projections from embryos treated with increasing concentrations of Peptide C and Peptide E are shown in Figures 5D,E, respectively.

#### 3.6.2 Peptide C but not peptide E induces a dose-dependent decrease in locomotion

The impact of peptide treatment on motor function was represented by the locomotion and swimming behavior of larvae, which was observed at 120 hpf under specific conditions. An epileptic seizure is usually caused by abnormal neuronal activity within the brain, and the total distance traveled by the zebrafish embryo increases as the embryos undergo spiraling, twitching, or uncontrolled jerking movements compared to the control (Zhao et al., 2015).

**FIGURE 6:**
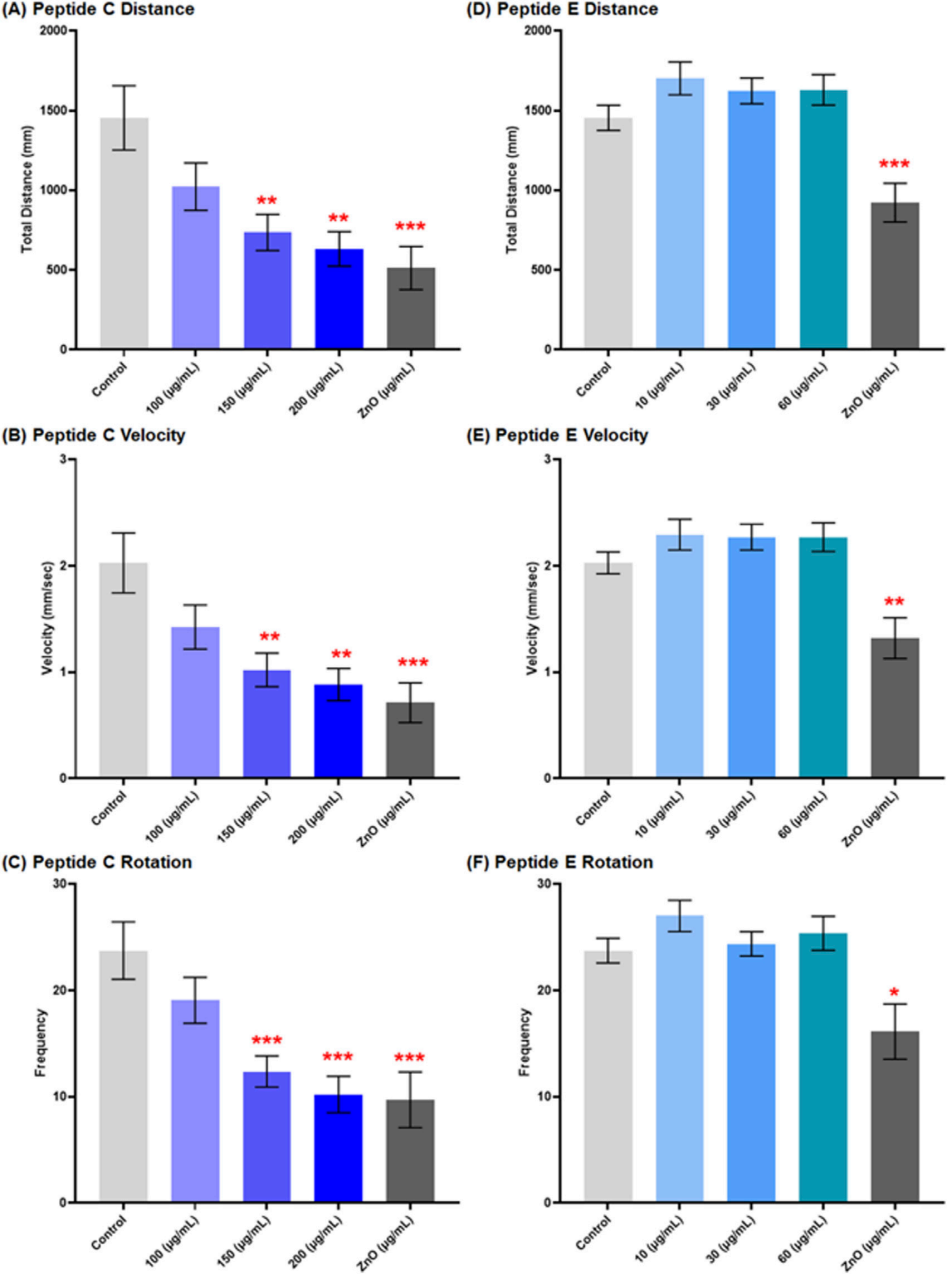
Effect of Peptides C and E on the locomotor activity of zebrafish embryos at 120 hpf. Zebrafish embryo movements were recorded using EthoVision XT software (Noldus Technology, Netherlands) under alternating light/dark cycles (2 min dark, 2 min light, 2 min dark, repeated) for a total duration of 12 minutes. A total of ten embryos were analyzed per treatment for Peptide C and fifteen for Peptide E. **(A–C)** Graphical representation of the effect of Peptide C on total distance moved **(A)**, velocity **(B)**, and total body rotation frequency **(C)**. **(D–F)** Graphical representation of the effect of Peptide E on total distance moved **(D)**, velocity **(E)**, and total body rotation frequency **(F)**. Data are presented as mean ± SEM. Statistical analysis was performed using Welch’s t-test to compare each treatment group to the negative control. Statistical significance: *p < 0.05, **p < 0.01, ***p < 0.001, and ****p < 0.0001 as compared to the negative control.

Peptide C treatment resulted in a concentration-dependent reduction in larvae locomotion. The mean distance moved in the control group was 1,454 mm ± 201.6, and at 100 μg/mL, it was 1,023 mm ± 148.5. Treatment with a higher concentration resulted in a significant reduction; for the 150 μg/mL group, the mean distance was 735.7 mm ± 113.5; for the 200 μg/mL group, 632.1 mm ± 108.1, and ZnO (positive control) (512.2 mm ± 135.0) (Figure 6A). A similar trend was observed for the calculated mean velocity. In the control group, the mean velocity was 2.03 mm/s ± 0.28, while for 100 μg/mL, it was 1.43 mm/s ± 0.21, and a significant reduction for the 150 (1.02 mm/s ± 0.16), 200 (0.89 mm/s ± 0.15), and ZnO 30 μg/mL (0.71 mm/s ± 0.19) (Figure 6B). In response to the sensory stimuli of dark-light alternate cycles, rotation frequencies for both counterclockwise (CCW) and clockwise (CW) directions were calculated. The total rotation frequency showed a concentration-dependent decrease with increasing Peptide C concentrations (Figure 6C) (Supplementary Figure S1A).

For Peptide E, the locomotion of zebrafish embryos was assessed after a 24-h treatment in different concentrations, starting at 96 hpf to 120 hpf. The results showed no significant changes in distance moved, a slight increase at 10 and 60 μg/mL, but a decrease at 30 μg/mL. Velocity results exhibited a slight increase at 10 μg/mL and subsequent decreases at 30 and 60 μg/mL compared to each other but showed an increase compared to the control. CCW rotation frequencies increased concentration-dependently at concentrations higher than the control, while CW rotation frequencies indicated a significant increase at 10 μg/mL, a decrease at 30 μg/mL, and an increase at 60 μg/mL. Total rotation frequencies increased compared to the control; however, there was an increase at 10 and 60 μg/mL, and 30 μg/mL indicated a decrease in total rotation (Figures 6D–F) (Supplementary Figure S1B).

#### 3.6.3 Peptide C and peptide E exhibit hepatotoxicity at moderate concentrations on zebrafish embryos

The hepatotoxicity assay was performed using the transgenic zebrafish line *(Fab10p: dsRed,* expressing red fluorescence in hepatocytes) strain. Zebrafish embryos were collected and treated with different concentrations of Peptide C, Peptide E, PTU (negative control), and ZnO (positive control). The liver size was calculated by imaging the embryos at 120 hpf. According to the timeline of the liver development in zebrafish, primary liver morphogenesis is completed at 48 hpf, and the liver is fully functional at 120 hpf (Al-Jamal et al., 2020; He et al., 2013; Chu and Sadler, 2009).

**FIGURE 7:**
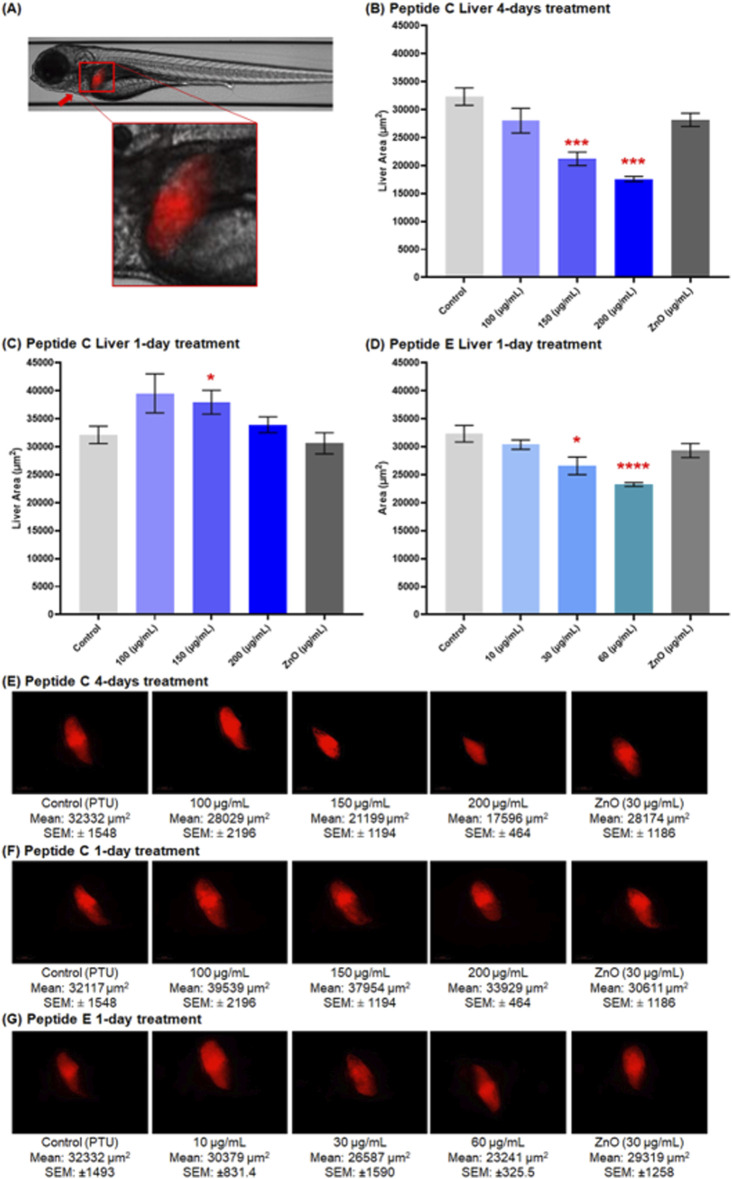
Effect of Peptides C and E on liver size in zebrafish embryos at 120 hpf. Liver images were captured at 20× magnification using the Vast Bioimager (Union Biometrica, Spain), and the liver area was analyzed using Danioscope Software (Noldus Technology, Netherlands). **(A)** Representative image of a zebrafish liver at ×5 magnification. **(B,C)** Graphical representation of liver area (µm^2^) following Peptide C treatment: **(B)** after 4-day incubation (n = 6), and **(C)** after 1-day incubation (n = 10). **(D)** Graphical representation of liver area after 1-day Peptide E treatment (n = 9). **(E–G)** Representative liver images showing red fluorescence in hepatocytes for: **(E)** Peptide C (4-day treatment), **(F)** Peptide C (1-day treatment), and **(G)** Peptide E (1-day treatment). Data are presented as mean ± SEM. Statistical analysis was performed using Welch’s t-test to compare each treatment group to the negative control. Statistical significance: *p < 0.05, **p < 0.01, and ***p < 0.001 as compared to the negative control.

Figure 7A presents a representative image of a zebrafish liver, illustrating the typical liver morphology used as a reference for evaluating potential hepatotoxic effects in treated groups. Peptide C treatment negatively affected the liver size. The size decreased in a concentration-dependent trend over a 4-day treatment period with peptide C (Figure 7B). However, after a 1-day treatment, the liver size increased compared to the control (Figure 7C).

For Peptide E, hepatotoxicity was assessed after 24-h treatment in different concentrations, starting at 96 hpf till 120 hpf. A significant decrease in liver size occurred at 30 and 60 μg/mL concentration, with a non-significant reduction at 10 μg/mL (Figure 7D). The overall trend indicated a concentration-dependent decrease in the liver area compared to the control (PTU) and ZnO (30 μg/mL). Representative liver images highlighting red fluorescence in hepatocytes are shown in Figures 7E–G. These include embryos treated with Peptide C for four days (Figure 7E), Peptide C for one day (Figure 7F), and Peptide E for one day (Figure 7G).

## 4 Discussion

The widespread use of antibiotics, derived initially from natural microbial products, has led to an alarming increase in antibiotic resistance and associated health risks, even in cases unrelated to microbial infections (Hutchings et al., 2019; Bassitta et al., 2022; Strömberg Celind et al., 2018; Elmahi et al., 2022). As an alternative, antimicrobial peptides (AMPs) have emerged as promising therapeutic agents due to their cationic, amphiphilic, and compact structures, which enable them to interact effectively with microbial membranes. Naturally produced by a wide range of organisms, including bacteria, fungi, plants, and animals, AMPs play a crucial role in innate immunity. However, despite their therapeutic potential, the practical application of AMPs presents significant challenges, even after successful *in silico* design and *in vitro* validation. Key concerns include assessing their toxicity in complex biological systems, determining their potential for organ-specific damage, understanding pharmacokinetics and stability in physiological environments, and translating effective *in vitro* concentrations to whole-organism models. Addressing these issues requires a robust pre-validation framework before progressing to targeted pathogen studies or cancer research to ensure safety and efficacy.

To evaluate these challenges, zebrafish embryos (*Danio rerio*) were used as an *in vivo* model due to their unique advantages. Their transparent nature allows for real-time monitoring of organ development and toxicity, their rapid embryonic development facilitates time-efficient screening, and their high genetic and physiological similarity to humans makes them a relevant system for studying drug pharmacodynamics and toxicology (Hussen et al., 2024; Adhish and Manjubala, 2023). Utilizing zebrafish embryos as an initial testing model provides valuable insights into systemic toxicity, developmental abnormalities, and the broader implications of engineered AMPs before advancing to more complex mammalian models.

In this study, two bioinspired short antimicrobial peptides (BSAMPs), Peptide C and Peptide E, were designed *in silico* for their pro-apoptotic properties and subsequently validated *in vitro* for their efficacy against colorectal cancer. Their anticancer activity was assessed using three distinct colorectal cancer models—SW620, SW480, and HCT116—representing different stages of tumor progression (Hashem et al., 2025). SW480 cells, derived from a primary adenocarcinoma, model early-stage colorectal cancer, whereas SW620 cells, originating from a metastatic site in the same patient, simulate late-stage, metastatic disease. HCT116 cells, known for their highly aggressive and poorly differentiated phenotype, serve as a model for advanced-stage colorectal carcinoma. The results demonstrated that both peptides effectively induced apoptosis in all three colorectal cancer models while exhibiting selective cytotoxicity, as they did not affect the viability of normal human colon epithelial cells (CCD841). This selective activity highlights their potential as targeted anticancer agents with minimal off-target toxicity.

To further investigate their systemic effects, the peptides were evaluated in zebrafish embryos to assess their safety, stability, and biodistribution. This model allowed for organ-specific toxicity assessment and the detection of developmental abnormalities not typically captured in standard *in vitro* assays (Teame et al., 2019; Ennerfelt et al., 2019). Survival analysis between 24- and 96-h post-fertilization (hpf), a critical period for organogenesis, revealed a concentration-dependent decrease in survival. Peptide C exhibited 100% mortality at concentrations above 200 μg/mL, whereas Peptide E reached this threshold at 150 μg/mL. The LC50 values were determined to be 162.2 μg/mL for Peptide C and 131.82 μg/mL for Peptide E, classifying both peptides as practically non-toxic according to the Fish and Wildlife Service’s Acute Toxicity Rating Scale (El-Harbawi, 2014). Interestingly, no morphological abnormalities were observed despite the reduction in survival at higher concentrations. However, Peptide E demonstrated lower tolerance, requiring shorter exposure times (24 h) compared to Peptide C, which was administered through accumulated incubation.

The distinct effects of Peptide E on cell viability suggest a mechanism beyond pro-apoptotic activity, possibly involving metabolic disruption (Hashem et al., 2025). This difference may explain the greater embryonic sensitivity to Peptide E compared to Peptide C. Given that Peptide C primarily induces programmed cell death, its mechanism likely involves selective apoptosis in cancer cells while sparing healthy tissues, leading to a more controlled biological response.

Organ-specific toxicity experiments revealed no significant effects for Peptide C at concentrations up to 200 μg/mL, whereas Peptide E showed no detectable toxicity at 10, 30, and 60 μg/mL. Cardiotoxicity assessments demonstrated a concentration-dependent decrease in heart rate and blood flow for both peptides, though Peptide E induced heart arrhythmia, suggesting a more significant impact on cardiac function (Wei et al., 2021). Neurodevelopmental toxicity analysis revealed that Peptide C significantly reduced axon length and impaired locomotion, while Peptide E showed inconsistent neurotoxic effects, necessitating further investigation. Hepatotoxicity assessments at 120 hpf indicated a concentration-dependent reduction in liver size for both peptides. Peptide C induced hepatomegaly, likely due to an inflammatory response, while Peptide E caused hepatocyte damage and liver necrosis, indicating greater hepatotoxic potential.

While Peptides C and E demonstrate promising anticancer potential, this study highlights their potential toxicological concerns at higher concentrations and prolonged exposure. These findings underscore the importance of thorough preclinical evaluations, particularly regarding their effects on cardiac, neurodevelopmental, and hepatic systems in zebrafish embryos, before advancing to mammalian models (Al-Jamal et al., 2020; Miyawaki, 2020). Future studies should investigate the detailed mechanisms of action of BSAMPs *in vivo*, particularly their impact on mitochondrial function and metabolic processes, determining optimal dosing strategies to balance therapeutic efficacy and safety.

Long-term exposure studies are needed to assess chronic effects beyond early development and evaluate their safety and efficacy in mammalian models before considering clinical applications (Amawi et al., 2021; Cabezas-Sáinz et al., 2020). Although zebrafish provide valuable preliminary insights, the physiological differences between zebrafish and mammals necessitate cautious interpretation of findings before extrapolating results to human applications.

Several factors must be addressed for successful clinical translation, including stability, delivery mechanisms, and immunogenicity. In silico analyses, a half-life of 954.61 s was predicted for Peptide C and 840.31 s for Peptide E, suggesting favorable stability. However, peptide-based therapies frequently face challenges with rapid protease degradation, limiting their bioavailability. Potential strategies to enhance stability include liposome encapsulation, nanoparticle-based formulations, and injectable hydrogels for controlled, localized release at tumor sites. These methods not only protect peptides from enzymatic degradation but also improve targeted delivery and therapeutic efficacy. Effective delivery mechanisms are crucial for ensuring that the peptides reach their intended targets while minimizing systemic toxicity. While our *in silico* immunogenicity analyses using ToxiPred and Antigenic web tools indicated minimal immunogenic risk, preclinical validation in mammalian models remains essential to ensure clinical safety.

## 5 Conclusion

Zebrafish embryos proved effective for evaluating BSAMPs designed *in silico*, enabling early assessment of safety, organ-specific toxicity, and systemic effects. Peptides C and E, selected for their *in vitro* anticancer activity against colorectal cancer cells, exhibited distinct toxicity profiles. Peptide C showed concentration-dependent mortality without significant morphological abnormalities, suggesting a moderate level of systemic toxicity. In contrast, Peptide E induced developmental toxicity along with cardiotoxicity and hepatotoxicity, indicating broader cytotoxic effects potentially involving mitochondrial and metabolic dysfunction.

While zebrafish offer important advantages as a rapid and cost-effective *in vivo* model, it is important to recognize their limitations, including the absence of a fully developed adaptive immune system and differences in metabolic pathways compared to mammals. Therefore, findings from zebrafish studies should be interpreted cautiously and complemented by validation in mammalian models.

Considering the relatively favorable safety profile of Peptide C, additional validation studies in rodent models are warranted. Overall, this study highlights the utility of zebrafish embryos in the early evaluation of peptide therapeutics and supports the continued development of BSAMPs as targeted anticancer agents, while emphasizing the need for further studies to refine dosing strategies, evaluate long-term safety, and confirm translational potential in more complex biological systems.

## Data Availability

The raw data supporting the conclusions of this article will be made available by the authors, without undue reservation.
