# Supplementary figures and images for: Organ-specific safety profile of bioinspired short antimicrobial peptides in zebrafish embryos
Source: Front Pharmacol. 2025 May 27;16:1593683. doi: 10.3389/fphar.2025.1593683 (PMC12149112; doi:10.3389/fphar.2025.1593683)

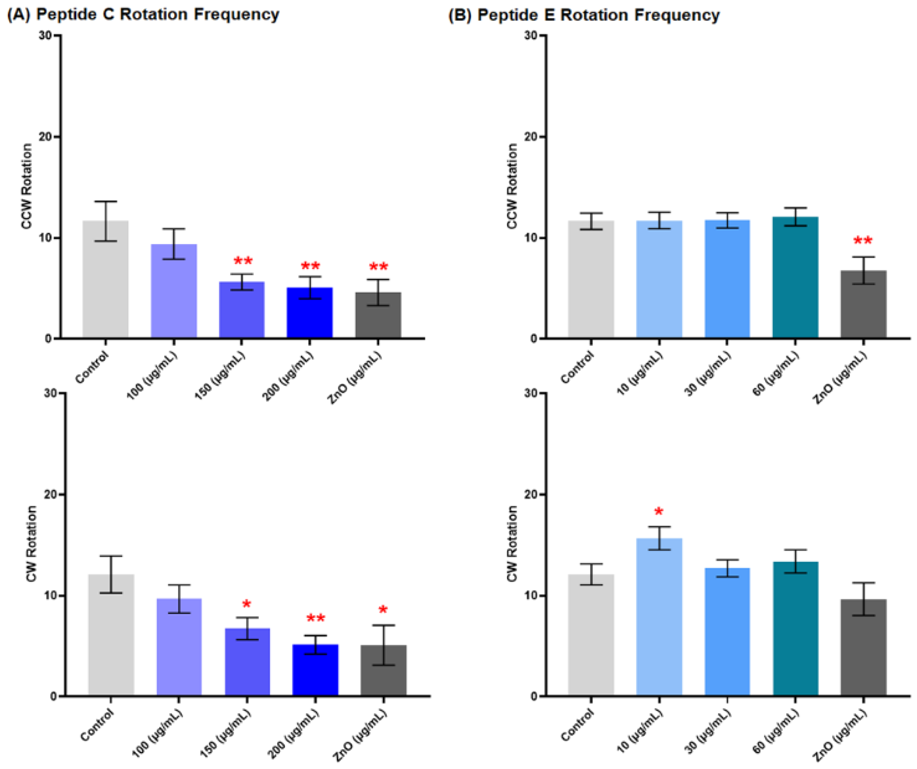

Supplement: Supplementary file 1 [file Image1.tif]
